# Risk Factors and Epidemiology of Coccidioidomycosis Demonstrated by a Case of Spontaneous Pulmonary Rupture of Cavitary Coccidioidomycosis

**DOI:** 10.1155/2016/8165414

**Published:** 2016-01-24

**Authors:** Amy A. Yau

**Affiliations:** Internal Medicine, William Beaumont Army Medical Center, 5005 North Piedras Street, El Paso, TX 79920, USA

## Abstract

A 31-year-old Hispanic male with no medical history was admitted for fevers, pleurisy, and cough after recent oral surgery and completing demolition and construction work in Juarez, Mexico. Imaging showed a 4.4 cm cavitary lesion and bilateral tree-in-bud opacities. Initial suspicion of bacterial infection confirmed with clinical improvement on culture specific antibiotics, but after discharge he returned with progression of symptoms and new dyspnea. Radiograph showed a pyopneumothorax. Chest computed tomography after thoracostomy showed worsening infiltrates and another cavitary lesion. Symptoms persisted despite addition of broad spectrum antibiotics. Surgical repair for persistent air leak was required. Weeks after discharge, cultures and serologies returned positive for* Coccidioidomycosis immitis*.* Coccidioides* species cause up to 30% of community-acquired pneumonia and incidental cavitary lesions in endemic regions. Symptoms are nonspecific yet usually involve fatigue, cough, and pleurisy. Most hosts have spontaneous resolution; however, certain demographics such as Hispanics and diabetics, later diagnosed in our patient, have higher morbidity. As seen with our patient, cavitary rupture and bronchopleural fistulas are rare occurring in 2.6% of cavitary lesions. High suspicion based on symptoms and host demographics is important to assist in early diagnosis and treatment to avoid and treat this common pathogen's presentations.

## 1. Introduction

Coccidioidomycosis is prevalent in the southwestern United States. However due to migration and travel, cases have been reported across the world and with increasing frequency. Data from the Centers for Disease Control demonstrate an increased incidence from 5.3 per 100,000 population in 1998 to 42.6 per 100,000 population in 2011 [1]. This approximates a 16% annual increased incidence. In endemic regions,* Coccidioides* species are responsible for 15–30% of community-acquired pneumonia [2]. This dimorphic fungus is found in soil; thus cases in endemic regions spike after rainy seasons (i.e., spring, end of summer) and natural or man-related activities that disturb the environment (i.e., dust storms, earthquakes, excavations, and construction).* Coccidioides* infection, also known as Valley Fever, was first described after multiple cases of patients became infected from* Coccidioides immitus* in the San Joaquin Valley area in California; however it is endemic throughout the desert southwest United States. A second species,* Coccidioidomycosis posadasii*, is genetically different but presents similarly and in the same endemic regions. As more cases appear, it is important for clinicians to have a high index of suspicion and understand full spectrum of disease and rare complications that can occur.

## 2. Case Presentation

A 31-year-old previously healthy male presented for two weeks of fevers, productive cough, and pleurisy. Symptom onset followed recent wisdom tooth extraction and travel to Juarez, Mexico, for construction work. He is a resident of El Paso, Texas. On admission he was febrile, and chest auscultation revealed left basilar crackles.

**Figure 1:**
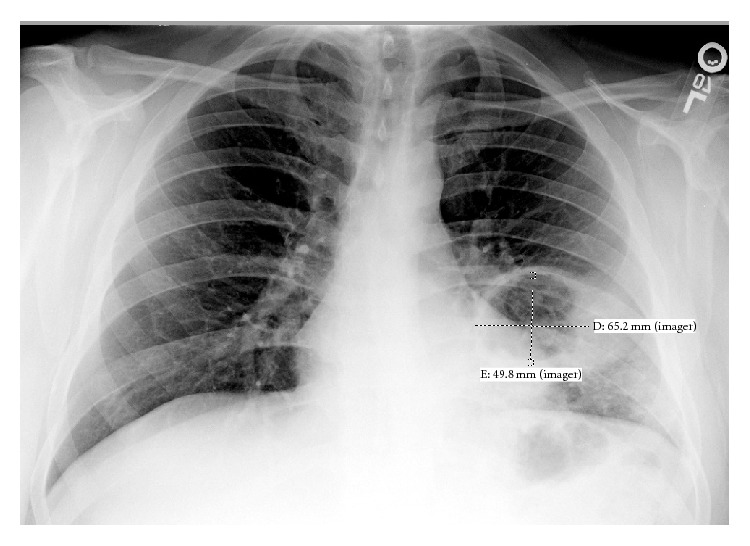
Chest X-ray on admission with 6.52 cm by 4.98 cm cavitary lesion.

**Figure 2:**
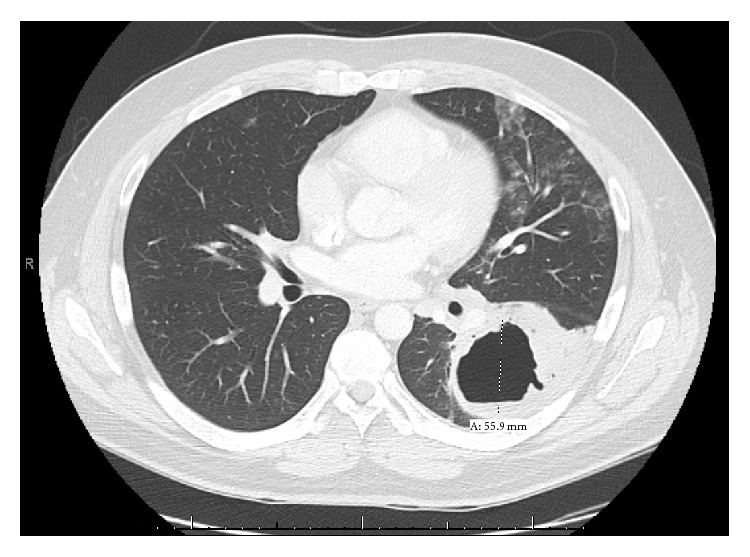
Chest computed tomography of left lower lobe superior segment cavitary lesion with air fluid level, largest diameter 5.59 cm.

His laboratory findings revealed a white blood cell count of 13.0 K/*μ*L, and chest radiography demonstrated a 4.4 cm cavitary lesion (Figure 1). Further evaluation via chest CT revealed an air fluid level (Figure 2), left hilar and mediastinal lymphadenopathy, and bilateral tree-in-bud and nodular opacities. Bronchoscopic evaluation did not reveal bronchial abnormalities, but cultures from lavage grew* Haemophilus influenza* and Group F* Streptococcus*. He was diagnosed with a necrotizing pneumonia and discharged with amoxicillin clavulanate after resolution of his fever and leukocytosis.

**Figure 3:**
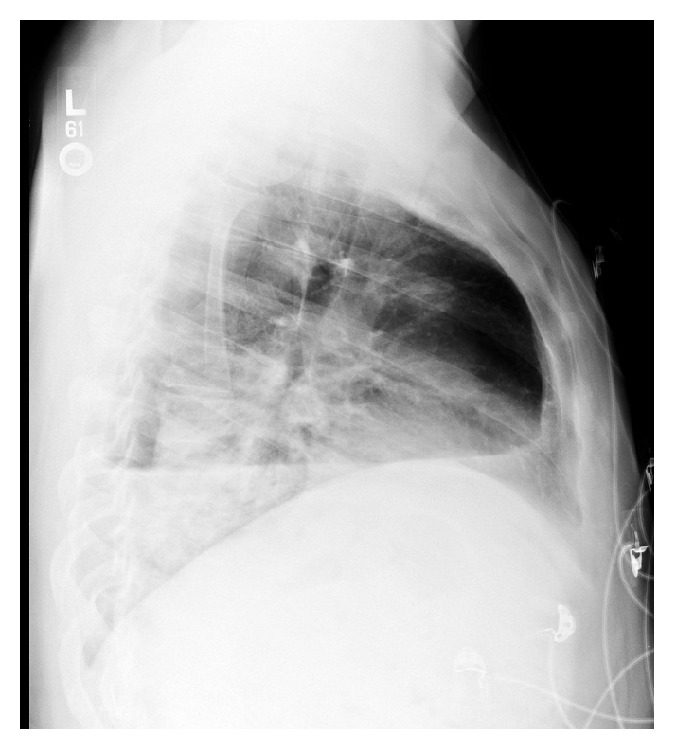
Chest X-ray on representation demonstrating pyopneumothorax.

Five days later he returned with recurrent fevers and dry cough. He complained of new acute dyspnea and worsening of pleurisy after a coughing fit. He denied arthralgias. He was febrile up to 103.0°F with otherwise stable vitals. Pulmonary exam was significant for absent breath sounds on the left, splinting, and midline trachea. There were no meningeal signs or skin lesions. His white blood cell count was 17.5 K/*μ*L. Chest radiograph on this admission showed left sided pyopneumothorax (Figure 3).

Our patient was readmitted and a chest tube placed. Repeat chest CT demonstrated worsening of disease via extension of pulmonary opacities and lymphadenopathy. Repeat respiratory cultures grew* Enterococcus cloacae*, but he did not respond to broad spectrum antibiotics and had persistent fever and leukocytosis. Multiple acid fast bacilli stains and cultures remained negative. Direct fluorescent assay for* Pneumocystis jirovecii*,* Cryptococcus* antigen, and galactomannan antigen for* Aspergillus* were negative.

Serology sent during his first hospitalization due to high suspicion returned ID-TP and ID-CF positive for* Coccidioidomycosis immitis* with titers 1 : 16 and 1 : 8, respectively. He was started on fluconazole but underwent superior segmentectomy due to persistent air leak. Interestingly segmented tissue did not stain positively for any organism, and over a month later, lavage and intraoperative pleural fluid cultures grew* Coccidioidomycosis immitis*. The patient demonstrated continued improvement and was treated with an extended course of fluconazole followed by local specialists.

## 3. Discussion

Our patient presented multiple confounders to his eventual diagnosis. Despite living and working in an endemic area, his history of oral surgery and response to antibiotics were red herrings to the culprit,* Coccidioidomycosis immitis*. Additionally, the dramatic nature of his disease prompted screening for dissemination, HIV, and quantitative immunodeficiencies. All were negative. During his hospital course, the patient was diagnosed with diabetes, and that coupled with his reported ethnicity of being Hispanic and geographical and environmental risk factors made coccidioidomycosis high on the differential. However as described below with the rarity of ruptured coccidioidomycosis pulmonary cavities and delay in diagnosis through serum and direct culture analysis, treatment can often be delayed.

Many patients with coccidioidal pneumonia are minimally troubled and do not seek medical treatment. Approximately 60% of infected patients will have nonspecific respiratory symptoms and significant fatigue one to three weeks after exposure [3]. Coccidioidomycosis can also manifest as nodules and cavitary disease. Asymptomatic pulmonary cavities are monitored [4]. Seen in 10–15% of patients with coccidioidomycosis, half of these cases demonstrate radiographic resolution without treatment [5]. The disease is considered noncontagious as inhaled arthroconidia, only 3–6 *μ*m in diameter, maneuver down terminal bronchioles and settle in alveoli [6]. There they proliferate into endospores and spherules, which are 20–60 *μ*m in diameter and too large to be brought up by expectoration [6]. Because of the periphery of the infection, pleuritic chest pain is often associated [7] as seen with our patient. Other symptoms include fatigue, arthralgias, erythema nodosum, and erythema multiforme, and more symptomatic patients go on to seek medical care. Disseminated coccidioidal mycosis is seen in less than five percent of cases and is typically seen in immunosuppressed groups, such as patients receiving high dose steroids or immunomodulators; patients with human immunodeficiency (HIV) infections; or patients with transplanted organs.

Other than immunosuppressed populations, there are other populations at increased risk for significant and symptomatic pulmonary disease. Patients with diabetes have a relative risk of 2.94 for development of cavitary disease compared to their nondiabetic counterparts [5, 8]. Poorly controlled diabetics carry an increased risk of relapsing and disseminated disease [8]. Complicated pulmonary disease, defined by nonresolving cavitation, cavitary rupture, and pyopneumothorax, requires surgical repair, and a quarter of these patients are diabetic [4, 9]. Epidemiologic data demonstrates Hispanics and African American individuals have a higher incidence of coccidioidal disease compared to their population [2]. Given most cases are asymptomatic, the increased case numbers suggest these demographics are more symptomatic and thus seek out medical care more than other demographics. Further data show Filipino and African American patients see more dissemination [1, 2, 10], and Hispanics and Native Americans have a higher age adjustment mortality compared to Caucasian counterparts [11].

These populations, specifically diabetics, may be at more risk due to their immunocompromised state compared to the general healthy population.* Coccidioidomycosis immitis* arthroconidia are phagocytized and induce a humoral immune response. S cell walls are hydrophobic and are hypothesized to release a protease that renders humoral mediated immune response negligible [7]. Dermal type IV hypersensitivity reactions and INF*γ* mediated endospore destruction support the importance of cell mediated immunity [7]. Interestingly, cellular immunity is inhibited in diabetic patients as hyperglycemia has been shown to inhibit maturation of dendritic cells and modify function of cytokines, such as IFN*γ*.

Despite known risk factors for severe disease, because of rarity of ruptured cavitary disease it is difficult to characterize risk factors for this population. Pleural effusions develop in 15% of patients infected [12], but pyopneumothoraces from spontaneous cavitary rupture are exceedingly rare. These episodes are described in healthy athletic males [13] and thought to occur in only 2.6% of cavitary coccidioidomycosis [9, 10]. Even more rare is the development of bronchopleural fistulas, which occur in one-eighth of these ruptured cavities [10].

Diagnosis requires understanding the manifestations of coccidioidomycosis and high suspicion especially in nonendemic regions. Although formation of antibodies may not be protective, the presence of serology can be used for diagnosis, and titers can prognosticate aggression of disease. Immunodiffusion to tube precipitin (ID-TP) is measurable within one to three weeks of onset, and immunodiffusion to complement fixating antigen (ID-CF) is measurable two to three weeks after inoculation and can be quantified. Immunoglobulin G (IgG) to CF titers more than 1 : 16 suggest severe disease [4]. Both methods are highly sensitive and specific, although repeat positive serologies increase their sensitivity; however results can take weeks to return depending on reference laboratories being used.

Gold standard for diagnosis is culture and/or species identification.* Coccidioides* species are cultured best from pleural biopsy with rare positivity on sputum and pleural fluid cultures. Fine needle aspiration (FNA) biopsies can yield spherule and rare mycelia forms [14], and bronchoalveolar lavage (BAL) will often demonstrate eosinophilia with positive mycelia seen on culture in 30–64% of specimens [15]. Cultured organisms are not specific and require confirmation with DNA probe and/or sequencing, which can take many weeks. Additionally, cultures must be handled carefully by certain laboratories given high risk for inhalation by laboratory workers of arthroconidia.

Treatment is recommended in symptomatic patients, patients with multiple comorbidities, high risk factors, or dissemination. Fluconazole is the azole of choice for most pneumonia and cavitary disease except for the pregnant patient, for whom amphotericin B is necessary [4]. Rare ruptured pulmonary cavities are treated surgically with lobectomy and decortication (AII recommendation). Despite these recommendations, there have been no randomized controlled trials showing if early treatment of uncomplicated pneumonia prevents morbidity or hastens recovery. Because of high suspicion and multiple risk factors for disseminated and presentation with complicated disease, our patient was started on fluconazole therapy while pending serum and culture confirmation. Perhaps increased awareness and economic impact analyses will prompt such research to help answer these questions.
